# Supplementary material for: Single-nucleus RNA-seq dissection of choroid plexus tumor cell heterogeneity
Source: EMBO J. 2024 Oct 31;43(24):6766–91. doi: 10.1038/s44318-024-00283-2 (PMC11649822; doi:10.1038/s44318-024-00283-2)
Supplement: Supplementary file 2 — Appendix [file 44318_2024_283_MOESM2_ESM.pdf]

Single-nucleus RNA-seq dissection of choroid plexus tumor cell heterogeneity

Anthony D. Hill[1,*], Konstantin Okonechnikov[2], Marla K. Herr[1,#], Christian Thomas[3], Supat Thongjuea[2], Martin Hasselblatt[3], and Annarita Patrizi[1,*]

[1]Schaller Research Group, German Cancer Research Center (DKFZ), 69120 Heidelberg, Germany
[2]Division of Pediatric Neurooncology, German Cancer Research Center (DKFZ) and German Cancer Consortium (DKTK), 69120 Heidelberg, Germany
[3] Institute of Neuropathology, University Hospital Münster, 48149 Münster, Germany

[#]Current address: Division of Molecular Neurobiology, Department of Medical Biochemistry and Biophysics, Karolinska Institute, 17177, Stockholm, Sweden

***Corresponding authors:**
Annarita Patrizi, PhD
German Cancer Research Center (DKFZ)
Tel: 004906221421551
a.patrizi@dkfz.de

Anthony D. Hill, PhD
German Cancer Research Center (DKFZ)
Tel: 004906221421551
a.hill@dkfz.de

Appendix Figure S1. Extracellular matrix (ECM)-receptor interaction pathway genes upregulated in choroid plexus tumor (CPT) epithelial cells

Appendix Table S1. Cell numbers and per cell average RNA, genes and percent mitochondrial reads by sample type, methylation profile and sample

Appendix Table S2. Expression of genes previously implicated in choroid plexus tumors

Appendix Table S3. Enriched KEGG pathways

Appendix Table S4. Nicotine addiction genes upregulated in choroid plexus tumors

Appendix Table S5. Tight junction component gene expression in choroid plexus tumors

Appendix Table S6. Select transporters upregulated in choroid plexus tumors

Appendix Table S7. Significant differentially expressed genes identified in non-epithelial choroid plexus tumor cell types

Appendix Table S8. Formalin-fixed paraffin-embedded (FFPE) human tissues

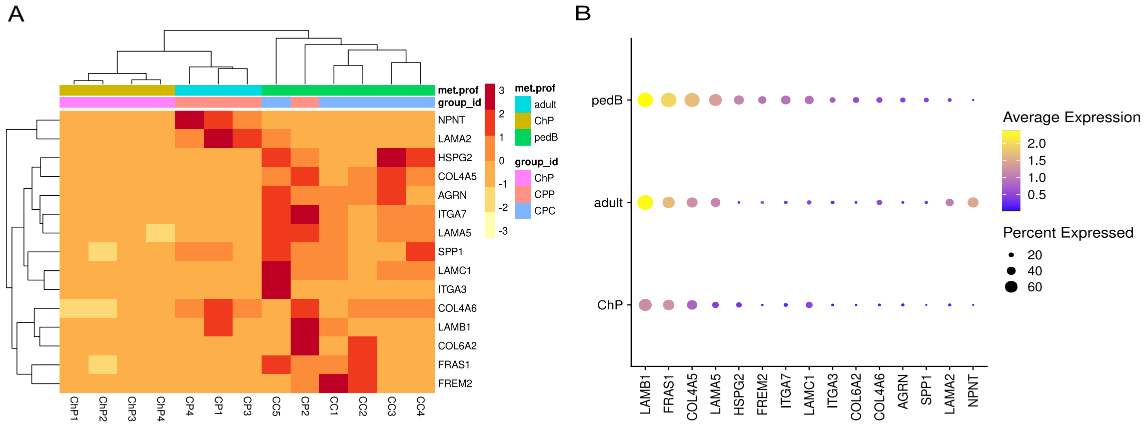

Appendix Figure 1. Extracellular matrix (ECM)-receptor interaction pathway genes upregulated in choroid plexus tumor (CPT) epithelial cells. **A**) Clustered heatmap of ECM-receptor interaction pathway gene expression in disease-free choroid plexus (ChP) and CPT samples. **B**) Dotplots of select ECM-receptor interaction pathway gene expression in epithelial lineage cells from ChP, adult profile tumors (adult) and pedB profile tumors (pedB).

Appendix Table S1. Cell numbers and per cell average RNA, genes and percent mitochondrial reads by sample type, methylation profile and sample

	n Cells	n RNA	n Genes	pct. mitochondrial
**Sample type**				
**ChP**	4732	6064	2644	2.54
**CPP**	5358	4430	2256	1.95
**aCPP**	1457	5670	2821	1.12
**CPC**	12364	4296	2408	1.01
**Meth. profile**				
**ChP**	4732	6064	2644	2.54
**adult**	1313	4185	2174	1.75
**pedB**	17861	4446	2412	1.25
**Sample name**				
**ChP1**	1620	5286	2514	1.73
**ChP2**	135	4075	1971	2.13
**ChP3**	1621	5650	2534	3.14
**ChP4**	1356	7685	2996	2.84
**CP1**	590	3984	2043	2.99
**CP2**	4045	4510	2283	2.02
**CP3**	271	3823	2302	0.84
**CP4**	452	4663	2266	0.67
**aCP1**	1273	5576	2874	1.28
**aCP2**	179	5383	2411	0.03
**CC1**	389	5776	2713	2.24
**CC2**	3100	4773	2602	1.40
**CC3**	638	1683	1319	0.31
**CC4**	4026	5183	2817	1.53
**CC5**	3692	3155	1927	0.07

Cell number (n Cells), average UMI count per cell (n RNA), average number of genes detected per cell (n Genes) and average percent mitochondrial reads (pct. mitochondrial) per sample type, methylation profile or sample listed in Table 1.

Appendix Table S2. Expression of genes previously implicated in choroid plexus tumors

Gene	L2FC (adult_Epithelial)	padj (adult_Epithelial)	L2FC (pedB_Epithelial)	padj (pedB_Epithelial)
NOTCH1	1.31*	1.22e-01	1.59*	2.66e-02
NOTCH2	0.26	6.87e-01	1.722*	1.122e-05
NOTCH3	0.51	3.65e-01	2.92*	1.78e-06
MYC	0.20	5.74e-01	-0.29	6.90e-01
TP53	0.13	8.69e-01	0.86	6.54e-02
RAD54L	0.08	9.06e-01	4.32*	3.37e-10
TAF12	-0.06	9.46e-01	0.70	1.24e-01
NFYC	-0.66	1.45e-01	-0.28	4.48e-01
PTEN	-0.20	7.75e-01	-0.55	3.04e-01

Expression changes for published CPT tumor genes in tumor epithelial cells from adult and pedB profile samples, relative to mean expression in disease-free choroid plexus epithelial cells (L2FC = log2 fold change). * = significant differentially expressed genes (log2 fold change > 1, adjusted p-value (padj) < 0.05).

Appendix Table S3. Enriched KEGG pathways

Pathway	Enrichment	n Genes	FDR
**KEGG pathway enrichment in adult**			
Neuroactive ligand-receptor interaction	2.9	20	.003
Nicotine addition	7.3	7	.004
ECM-receptor interaction	4.1	11	.006
Glutamatergic synapse	3.6	12	.007
Proteoglycans in cancer	2.7	17	.010
Rap1 signaling pathway	2.5	17	.016
Circadian entrainment	3.6	10	.016
Taste transduction	2.1	6	.020
PI3K-Akt signaling	2.1	21	.037
**KEGG pathway enrichment in pedB**			
microRNAs in cancer	2.1	46	.000
MAPK signaliing pathway	1.8	66	.000
Cell cycle	2.0	37	.001
ECM-receptor interaction	2.3	26	.002
Nicotine addition	3.1	13	.003
Calcium signaling pathway	1.7	49	.004
Pathways in cancer	1.4	97	.004
Morphine addiction	2.1	24	.007
Notch signaling pathway	2.4	18	.007
Th1 and Th2 cell differentiation	2.3	20	.008
Circadian entrainment	2.0	25	.007
PI3K-Akt signaling pathway	1.5	65	.009
Neuroactive ligand-receptor interaction	1.6	49	.010
Oxytocin signaling pathway	1.8	34	.010
cAMP signaling pathway	1.6	42	.015
Oocyte meiosis	1.8	30	.015
Progesterone-mediated oocyte maturation	1.9	25	.015
Relaxin signaling pathway	1.8	30	.015
Amphetamine addiction	2.2	17	.015
Ras signaling pathway	1.6	45	.018
Rap1 signaling pathway	1.6	44	.018
Axon Guidance	1.6	41	.018
Cocaine addition	2.4	13	.018
Human papillomavirus infection	1.5	59	.018
Breast cancer	1.7	31	.018
Growth hormone synthesis secretion and action	1.8	27	.021
Protein digestion and absorption	1.9	22	.021
Endocrine resistance	1.8	23	.025
Alcoholism	1.8	23	.025
GABAergic synapse	1.9	20	.030
Renin secretion	2.1	16	.031
Chemokine signaling pathway	1.6	32	.031

Focal adhesion	1.5	42	.031
Apoptosis	1.6	29	.03
Cushing syndrome	1.6	31	.031
Chemical carcinogenesis receptor activation	1.6	34	.035
Aldosterone synthesis and secretion	1.8	21	.042
Osteoclast differentiation	1.7	25	.043
Insulin secretion	1.7	25	.045
Platelet activation	1.7	26	.047
cGMP-PKG signaling pathway	1.5	33	.049
Cytokine-cytokine receptor interaction	1.5	33	.049

KEGG pathways enriched (FDR < 0.05, fold change ≥ 1), fold enrichment, number of upregulated pathway genes and FDR in choroid plexus tumors with adult or pedB methylation profiles.

Appendix Table S4. Nicotine addiction genes upregulated in choroid plexus tumors

Gene	Description
**CACNA1A**	Voltage gated calcium channel subunit
**GABRB1**	GABA receptor subunit
**GRIA3**	Ionotropic glutamate receptor subunit
**GRIA2**	Ionotropic glutamate receptor subunit
**GABRA2**	GABA receptor subunit
**GRIN2A**	Ionotropic glutamate receptor subunit
**GRIN2C**	Ionotropic glutamate receptor subunit
**CANCA1B**	Voltage gated calcium channel subunit
**GABRB2**	GABA receptor subunit
**GABRB3**	GABA receptor subunit
**GABRD**	GABA receptor subunit
**GABRG2**	GABA receptor subunit
**GABRR2**	GABA receptor subunit
**GABRQ**	GABA receptor subunit
**GABRE**	GABA receptor subunit
**GRIN2D**	Ionotropic glutamate receptor subunit

List of genes from the Nicotine addiction KEGG pathway upregulated in adult profile tumors only (blue), adult and pedB tumors (magenta) or pedB only (red).

Appendix Table S5. Tight junction component gene expression in choroid plexus tumors

Gene	L2FC (adult_Epithelial)	padj (adult_Epithelial)	L2FC (pedB_Epithelial)	padj (pedB_Epithelial)
CLDN5	-0.19	6.47e-01	-3.61*	5.38e-04
CLDN2	-1.19	0.87e-01	-5.17*	2.47e-18
CLDN1	0.23	6.87e-01	-1.99*	9.15e-03
OCLN	-0.82	1.56e-01	-2.39*	7.90e-08
JAM3	-0.44	4.54e-01	-0.87	5.38e-02
AFDN	-0.56	3.06e-01	0.16	7.44e-01
TJP1	-0.46	4.35e-01	0.60	2.17e-01

Expression changes for selected tight junction proteins in tumor epithelial cells from adult and pedB profile samples, relative to mean expression in disease free choroid plexus epithelial cells (L2FC = log2 fold change). * = significant differentially expressed genes (log2 fold change > 1, adjusted p-value (padj) < 0.05).

Appendix Table S6. Select transporters upregulated in choroid plexus tumors (CPT)

Gene	Transport function	L2FC (adult_ Epithelial)	padj (adult)	L2FC (pedB_ Epithelial)	padj (pedB)
SLC1A7	glutamate	6.04*	6.85e-06	9.71*	4.29e-19
SLC16A8	monocarboxylates	4.94*	7.57e-06	7.22*	3.37e-16
SLC8A1	sodium calcium	3.24*	3.00e-03	6.15*	4.95e-13
SLC24A3	Sodium potassium calcium	2.06*	7.74e-02	5.09*	1.63e-06
SLC25A48	acyl carnitine (mitochondrial)	1.72*	9.03e-03	2.77*	3.61e-08
SLC4A7	sodium bicarbonate	-0.08	9.18e-01	2.70*	6.16e-05
SLC9B2	sodium hydrogen	0.53	3.69e-01	2.69*	2.01e-10
SLC2A1	Glucose	-0.17	8.17e-01	2.30*	2.29e-05
SLC7A1	cysteine/glutamate antiport	-0.64	2.92e-01	2.05*	2.26e-04
SLC7A11	cysteine/glutamate antiport	-0.41	4.89e-01	1.93*	1.08e-06
SLC4A8	sodium bicarbonate	0.57	3.25e-01	1.59*	2.21e-04
SLC7A6	basic amino acids	0.13	8.73e-01	1.57*	1.29e-03
SLC35E2B	nucleotide sugar	0.00	9.99e-01	1.54*	1.96e-03
SLC35E3	carbohydrate derivative	0.23	7.44e-01	1.54*	1.96e-03
SLC39A10	zinc	0.67	2.57e-01	1.49*	1.39e-03
SLC13A3	sodium dicarboxylate	0.19	7.96e-01	1.42*	4.83e-03
SLC35G2	nucleotide sugar	0.46	4.62e-01	1.34*	1.48e-02
SLC1A3	glutamate	2.55*	1.60e-02	1.33*	6.94e-02
SLC16A2	thyroid hormone	0.82	1.34e-01	1.29*	1.65e-03
SLC3A2	leucine glutamine	1.30*	5.60e-02	1.23*	2.09e-02
SLC5A6	biotin sodium	-0.16	8.29e-01	1.13*	1.50e-02
SLC25A25	ATP inorganic phosphate	0.27	6.83e-01	1.11*	2.55e-02
SLC39A8	zinc	1.84*	3.21e-02	0.41	5.34e-01

Fold expression changes for solute transporter genes upregulated in CPT epithelial cells with adult (cyan), pedB (red) or both methylation profiles (magenta). L2FC = log2 fold change of adult or pedB epithelial cells relative to disease-free ChP epithelial cells), padj = adjusted p-value. * indicates significant DEGs (L2FC > 1, adjusted p-value < 0.05).

Appendix Table S7. Significant differentially expressed genes identified in non-epithelial choroid plexus tumor (CPT) cell types

Cell type	Upregulated adult	Upregulated pedB	Downregulated adult	Downregulated pedB
**Macrophage**	13	45	15	111
**Endothelial**	15	210	10	359
**Mesenchymal**	17	127	5	125

Number of significant (adjusted p value < 0.05) upregulated (log2 fold change > 1) and downregulated (log2 fold change <-1) genes identified by pseudobulk DEG analysis of Macrophage, Endothelial or mesenchymal cell gene expression in tumors with adult and pedB methylation profiles.

Appendix Table S8. Formalin-fixed paraffin-embedded (FFPE) human tissues.

Sample ID	Diagnosis
**N290_23**	CPP
**N394_23**	CPP
**N615_23**	CPP
**N1059_23**	CPP
**N2931_23**	CPP
**N4388_23**	CPP
**N2623_22**	CPC
**N3575_23**	CPC
**N4606_22**	CPC

Samples IDs and histological diagnoses of CPT samples used for immunohistochemistry. CPP, choroid plexus papilloma; CPC, choroid plexus carcinoma.
