# Supplementary figures and images for: Single-nucleus RNA-seq dissection of choroid plexus tumor cell heterogeneity
Source: EMBO J. 2024 Oct 31;43(24):6766–91. doi: 10.1038/s44318-024-00283-2 (PMC11649822; doi:10.1038/s44318-024-00283-2)

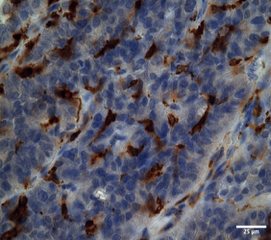

Supplement: Supplementary file 6 — Source data Fig. 6 [file 44318_2024_283_MOESM6_ESM.zip › Figure6/6D/SD_6D_CPCiba1.tiff]

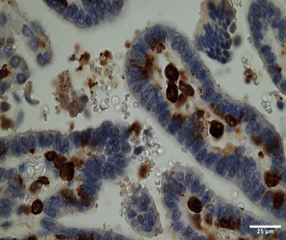

Supplement: Supplementary file 6 — Source data Fig. 6 [file 44318_2024_283_MOESM6_ESM.zip › Figure6/6D/SD_6D_CPPiba1.tiff]
