# Supplementary material for: Single-nucleus RNA-seq dissection of choroid plexus tumor cell heterogeneity
Source: EMBO J. 2024 Oct 31;43(24):6766–91. doi: 10.1038/s44318-024-00283-2 (PMC11649822; doi:10.1038/s44318-024-00283-2)
Supplement: Supplementary file 7 — Expanded View Figures [file 44318_2024_283_MOESM7_ESM.pdf]

Expanded View Figures

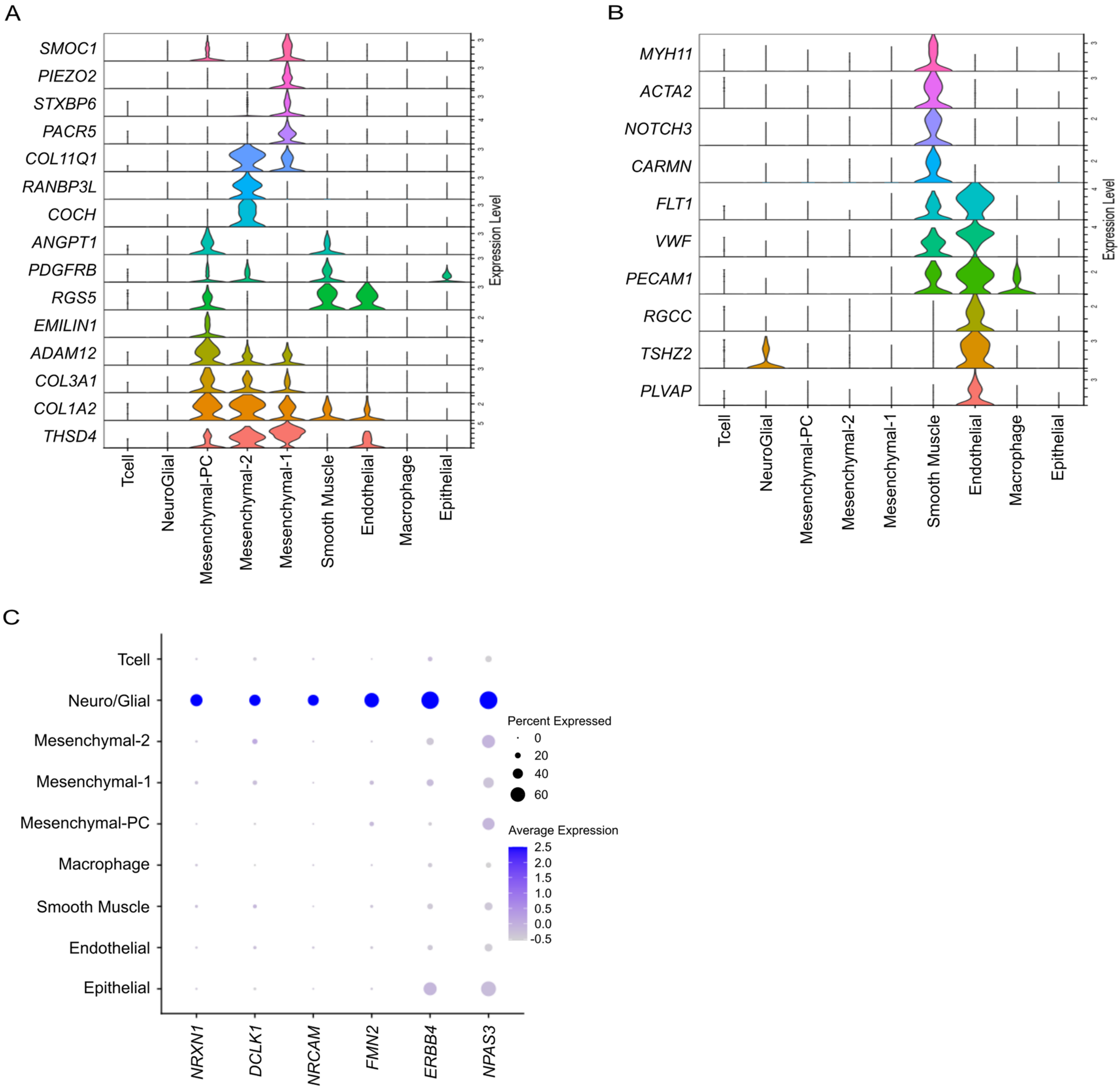

Figure EV1. Cellular composition of disease-free human choroid plexus (ChP) and choroid plexus tumors.

(**A**) Violin plots showing mesenchymal cell marker expression in disease-free human ChP. (**B**) Violin plots of endothelial and smooth muscle cell marker expression in disease-free human ChP. (**C**) Dotplot of neuronal and glial cell markers in human disease-free human ChP ($n = 4$ biological replicates).

© The Author(s)

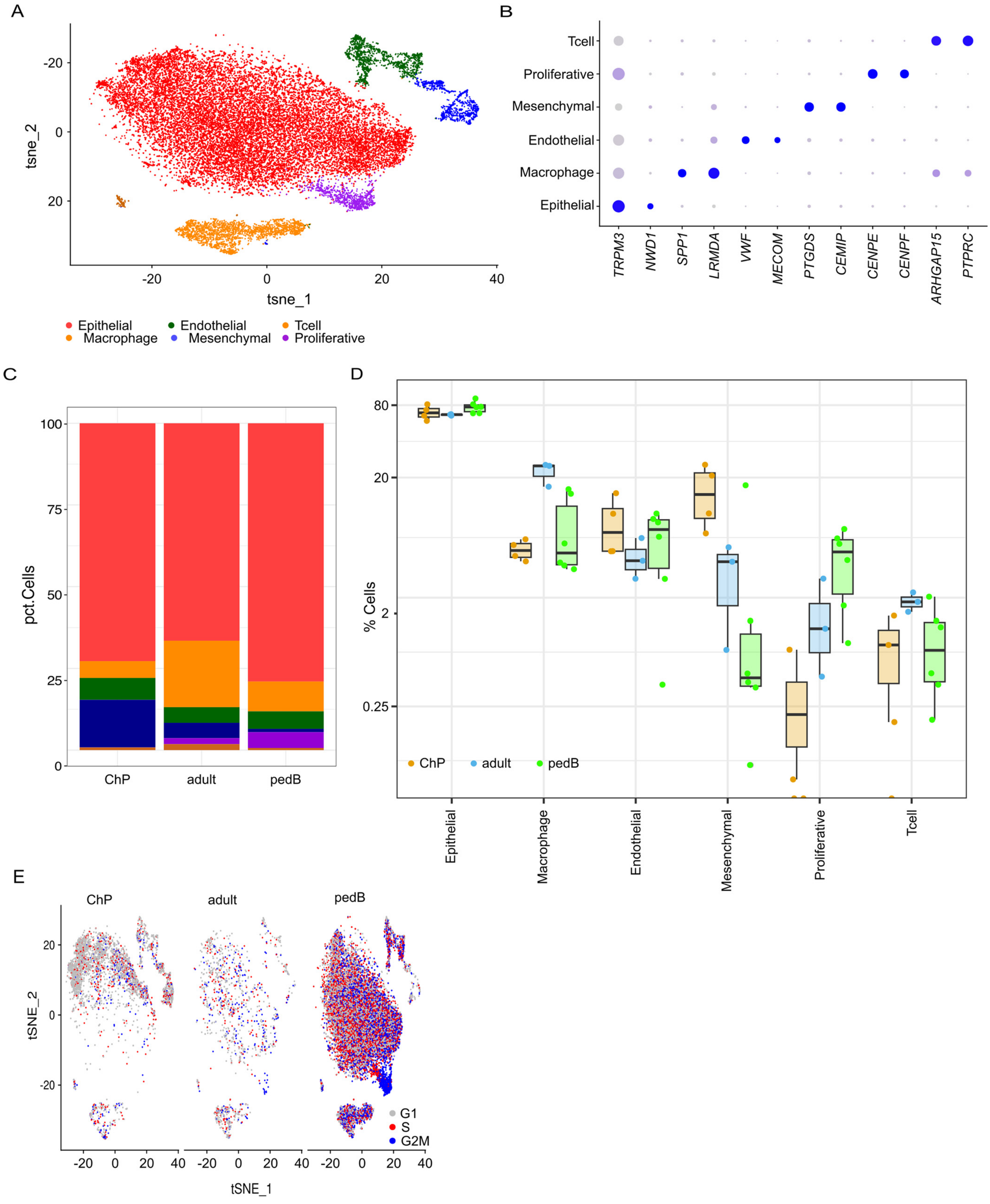
A
tsne_2
tsne_1
Epithelial
Endothelial
Tcell
Macrophage
Mesenchymal
Proliferative
B
Tcell
Proliferative
Mesenchymal
Endothelial
Macrophage
Epithelial
TRPM3
NWD1
SPP1
LRMDA
VWF
MECOM
PTGDS
CEMIP
CENPE
CENPF
ARHGAP15
PTPRC
C
pct.Cells
ChP
adult
pedB
D
% Cells
ChP
adult
pedB
Epithelial
Macrophage
Endothelial
Mesenchymal
Proliferative
Tcell
E
ChP
adult
pedB
tSNE_2
tSNE_1
G1
S
G2M

 © The Author(s)

Figure EV2. Cellular composition of choroid plexus tumors (CPTs).

(**A**) Cell clustering and dimension reduction of the entire batch corrected dataset of disease-free choroid plexus (ChP) and CPTs. (**B**) Dotplot of low *p*-value cluster specific gene expression in each cell cluster (Wilcoxon rank sum test-Seurat). (**C**) Relative cell number of major ChP cell types in CPT samples with adult or pedB methylation profiles, as well as disease-free ChP. Boxplots indicate 25th through 75th percentile, bold black line indicates median. Whiskers show range of data. (**D**) Percent of major cell types identified in each disease-free human ChP (gold, $n = 3$ biological replicates) or CPT sample, grouped by methylation (adult profile, blue; pedB profile, green; $n = 27$ and 12 biological replicates, respectively). (**E**) Cell cycle scoring by methylation profile and cell type. tSNE projections of cells in G1 (gray), S (red), or G2M (blue) phase of the cell cycle for disease-free ChP (left panel), adult profile CPT (middle panel), or pedB profile CPT (right) samples. (gold, $n = 3$ biological replicates), adult (blue, $n = 27$ biological replicates) and pedB (green, $n = 12$ biological replicates).

A

Gene	L2FC (adult_Epithelial)	padj (adult_Epithelial)	L2FC (pedB_Epithelial)	padj (pedB_Epithelial)
**STIL**	1.47	6.17E-15	3.80	2.27E-13
**PROM1**	3.01	0.04	4.82	5.10E-09
**BAIAP3**	2.75	4.22E-03	0.46	0.50
**WNT2B**	3.22	8.36E-08	1.95	7.60E-05
**WNT5B**	7.09	6.17E-15	9.63	1.18E-35
**GNB3**	6.19	1.15E-06	9.44	3.23E-19

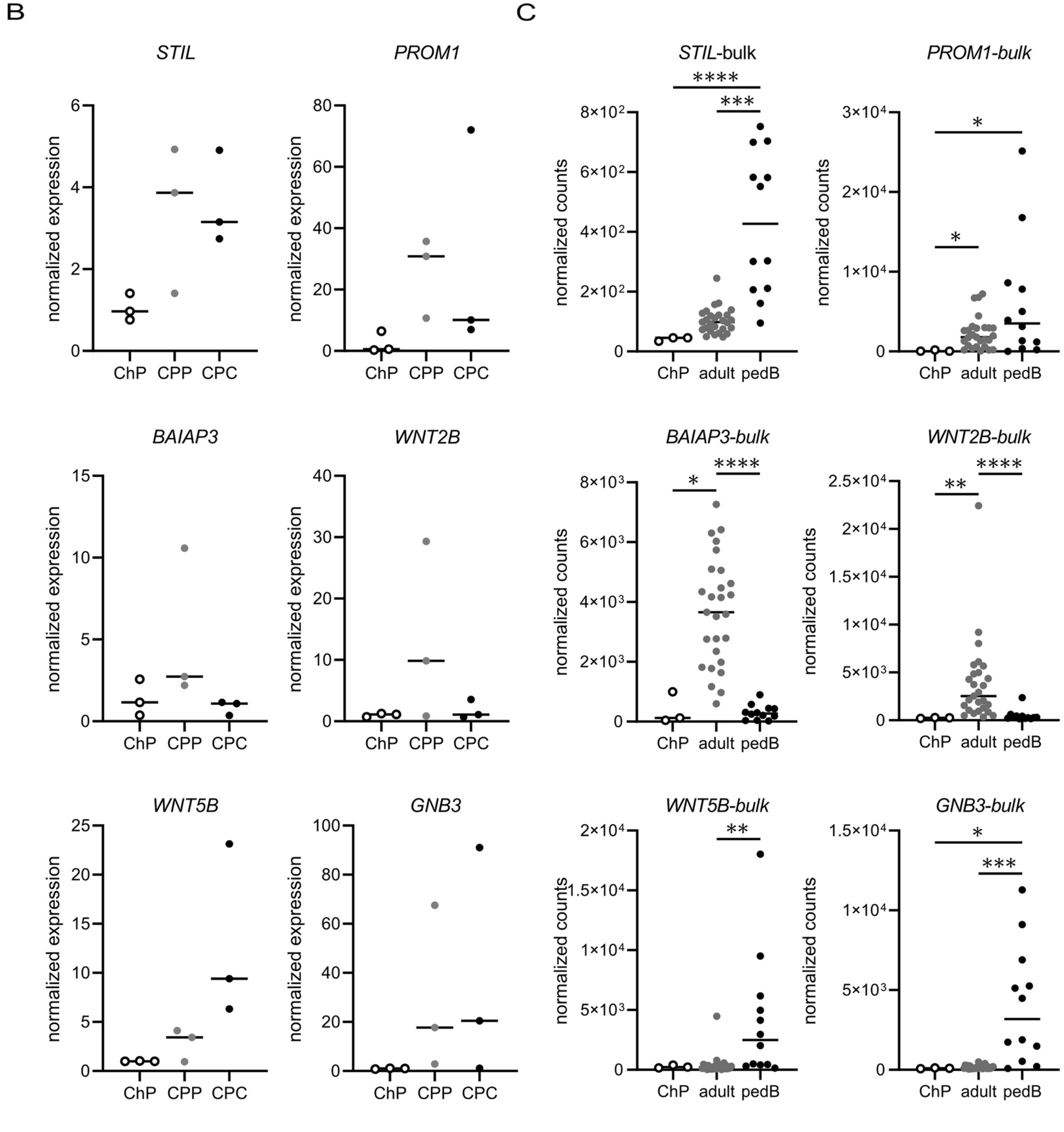

 © The Author(s)

Figure EV3. Expression of select differentially expressed genes identified in single nucleus (sn) analysis characterizing choroid plexus tumor (CPT) epithelial cells.

(**A**) Expression fold change and adjusted *p*-values for selected genes in pseudo-bulk analyzed snRNAseq data for adult and pedB profile epithelial cells. L2FC = log2 fold (Wald test-DESeq2). (**B**, **C**) Relative mRNA expression levels of candidate genes preferentially expressed in CPT epithelial cells (top row), candidate genes preferentially represented in low-risk CPT epithelial cells (middle row) and candidate genes preferentially expressed in high-risk CPT epithelial cells (bottom row). Expression analysis was performed by RT-qPCR (**B**) or by normalizing counts from published bulk sequencing (**C**). ChP, disease-free choroid plexus; CPP, papilloma; CPC, carcinoma; $n = 3$ biological replicates (3 technical replicates per sample) (**A**); see Thomas et al, 2021 for data information (**B**). Bars represent median. Kruskal–Wallis test adjusted *p*-values: *$p \leq 0.05$, **$p \leq 0.01$, ***$p \leq 0.001$, ****$p \leq 0.0001$ (*STIL1*-bulk ChP vs. pedB $p < 0.0001$, adult vs pedB $p = 0.0002$; *PROM1*-bulk ChP vs. adult $p = 0.0452$, ChP vs. pedB $p = 0.0122$; *BAIAP3*-bulk ChP vs. adult $p = 0.0177$, adult vs. pedB $p < 0.0001$; *WNT2B*-bulk ChP vs. adult $p = 0.0098$, adult vs. pedB $p < 0.0001$; *WNT5B*-bulk adult vs. pedB $p = 0.0016$; *GNB3*-bulk ChP vs. pedB $p = 0.0154$, adult vs. pedB $p = 0.0003$).

© The Author(s)

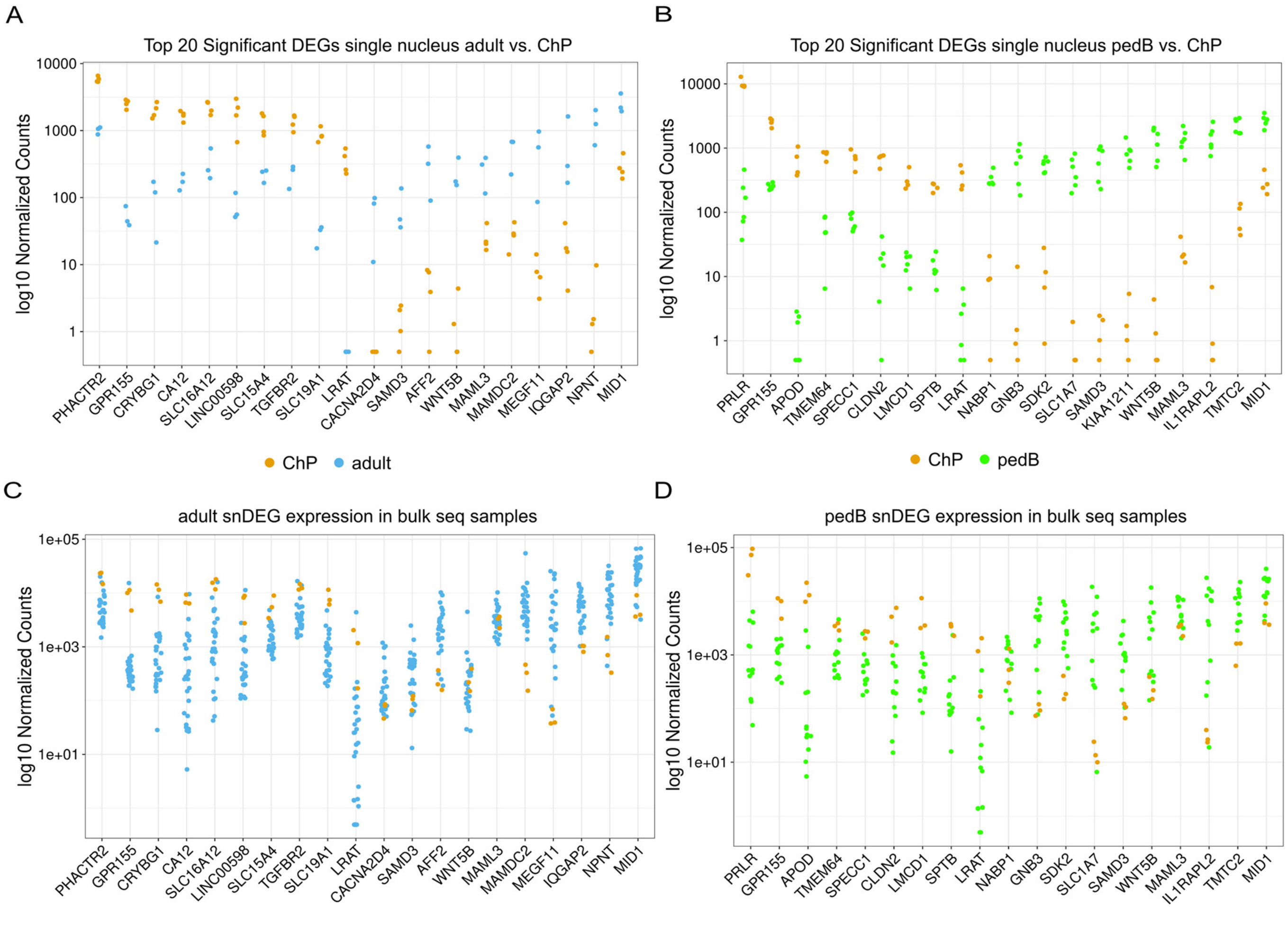

E

Gene	L2FC (sn_adult)	L2FC (bs_adult)	Gene	L2FC (sn_pedB)	L2FC (bs_pedB)
**PHACTR2**	-2.4*	-1.5*	**PRLR**	-5.7*	-4.8*
**GPR155**	-5.5*	-2.8*	**GPR155**	-3.3*	-2.4*
**CRYBG1**	-4.1*	-3.0*	**APOD**	-8.8*	-4.5*
**CA12**	-3.1*	-2.1	**TMEM64**	-3.7*	-1.2*
**SLC16A12**	-2.6*	-1.9	**SPECC1**	-3.2*	-1.7*
**LINC00598**	-4.5*	-2.1*	**CLDN2**	-5.1*	-3.1*
**SLC15A4**	-2.5*	-1.7*	**LMCD1**	-4.1*	-3.4*
**TGFBR2**	2.5*	-1.2*	**SPTB**	-4.2*	-2.8*
**SLC19A1**	-4.8*	-2.8*	**LRAT**	-7.8*	-1.3
**LRAT**	-10.4*	-0.4	**NABP1**	4.7*	0.2
**CACNA2D4**	10.11*	0.7	**GNB3**	9.4*	5.2*
**SAMD3**	5.5*	1.0	**SDK2**	6.1*	3.6*
**AFF2**	5.6*	3.2*	**SLC1A7**	9.7*	7.8*
**WNT5B**	7.1*	0.2	**SAMD3**	8.8*	3.3*
**MAML3**	3.5*	0.1	**KIAA1211**	8.5*	4.3*
**MAMDC2**	3.9*	4.3*	**WNT5B**	9.6*	3.5*
**MEGF11**	6.1*	6.6*	**MAML3**	6.0*	1.0*
**IQGAP2**	5.3*	2.4*	**IL1RAPL2**	9.2*	7.9*
**NPNT**	8.3*	3.4*	**TMTC2**	4.5*	2.8*
**MID1**	3.1*	2.1*	**MID1**	3.16*	1.4*

 © The Author(s)

Figure EV4. Expression of 20 top differentially expressed genes in each methylation tumor profile.

(**A–D**) Differentially expressed gene (DEG) expression in single nucleus (sn) and bulk sequencing (bs) samples. Normalized counts for 20 most significant DEGs for adult vs. disease-free choroid plexus (ChP) (**A**, **C**) or pedB vs. ChP (**B**, **D**) in each sn (**A**, **B**) or bs (**C**, **D**) library. (**E**) Expression fold change and adjusted *p*-values for genes graphed in (**A–D**) above. L2FC = log2 fold. * indicates significant DEGs (adjusted p-value < 0.05, log2 fold change >1 or <−1, Wald test-DESeq2).

© The Author(s)

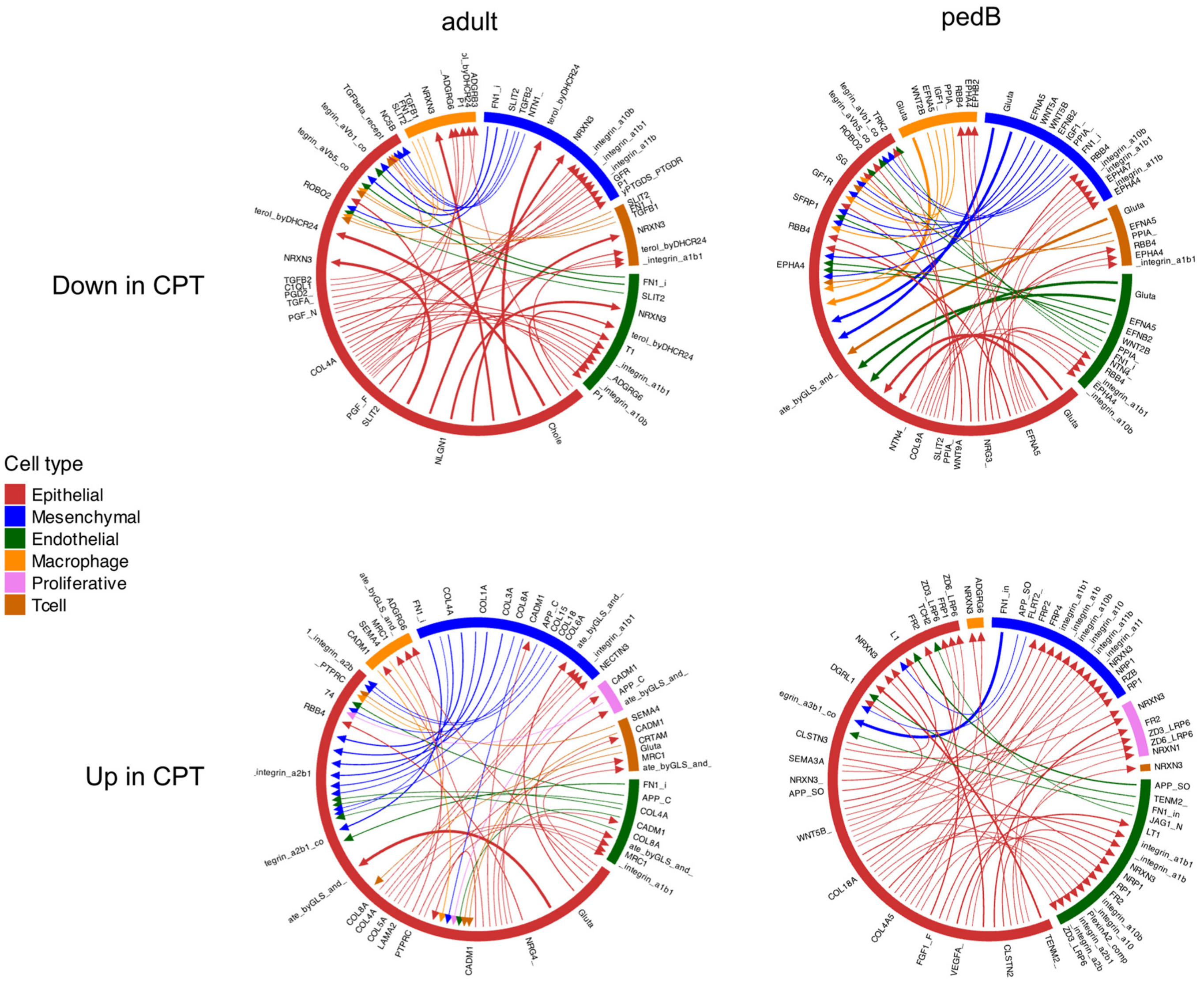

Figure EV5. Cell–cell interactions involving epithelial lineage cell differentially expressed genes in disease-free choroid plexus (ChP), adult, and pedB tumor samples.

Circos interaction diagrams of top interactions in ChP (top row) or tumors (bottom row, adult on left, pedB on right). Interactions are limited to genes significantly downregulated in adult and pedB (top row) or upregulated in adult and pedB (bottom row).

 © The Author(s)
